# Validation of 10-Minute Delayed Hepatocyte Phase Imaging with 30° Flip Angle in Gadoxetic Acid-Enhanced MRI for the Detection of Liver Metastasis

**DOI:** 10.1371/journal.pone.0139863

**Published:** 2015-10-07

**Authors:** Dahye Lee, Eun-Suk Cho, Dae Jung Kim, Joo Hee Kim, Jeong-Sik Yu, Jae-Joon Chung

**Affiliations:** 1 Department of Radiology, Yonsei University College of Medicine, Gangnam Severance Hospital, Seoul, Korea; 2 Department of Radiology, CHA University, CHA Bundang Medical Center, Seongnam-si, Korea; University of Chicago, UNITED STATES

## Abstract

**Objectives:**

To compare 10-minute delayed hepatocyte phase imaging using a 30° flip angle (10min-FA30) and 20-minute hepatocyte phase imaging using a 10° FA (20min-FA10) in gadoxetic acid-enhanced MRI of patients with possible liver metastases, regarding lesion-to-liver contrast-to-noise ratio (CNR) and focal hepatic lesion (FHL) detection to evaluate whether 10min-FA30 would be superior to 20min-FA10.

**Materials and Methods:**

Eighty-three patients with 248 liver metastases and 78 benign FHLs who underwent gadoxetic acid-enhanced MRI with 10min-FA30 and 20min-FA10 were enrolled. Lesion-to-liver CNRs were compared between the two image groups. Two radiologists independently assessed the presence of FHLs using a four-point scale and detection sensitivity was calculated.

**Results:**

The mean CNR for liver metastases on the 10min-FA30 (248.5 ± 101.6) were significantly higher than that of the 20min-FA10 (187.4 ± 77.4) (p < 0.001). The mean CNR difference between the two image groups was 61.2 ± 56.8. There was no significant difference in detection sensitivity of FHLs for two readers between 10min-FA30 (mean 97.7%) and 20min-FA10 (mean 97.9%), irrespective of the lesion size or malignancy.

**Conclusion:**

10min-FA30 yielded higher CNR with similar sensitivity compared to 20min-FA10. This finding indicates that 10min-FA30 can potentially replace 20min-FA10 with higher diagnostic performance and save 10 minutes of time.

## Introduction

Gadoxetic acid (Gd-EOB-DTPA) is a liver-specific magnetic resonance imaging (MRI) contrast agent that is widely used for both dynamic and hepatocyte-specific imaging [1]. This compound is taken up continuously by hepatocytes 1 minute after contrast administration, and increases the signal intensity of the liver parenchyma on the delayed phase imaging. Hepatocyte phase imaging (HPI) obtained at 20 minutes after injection of gadoxetic acid [2–5] has been proven to improve detection of focal hepatic lesions (FHLs), including liver metastases [6–12].

Several studies have shown that the time delay for HPI after injection of gadoxetic acid can be decreased less than 20 minutes. Many studies have shown that 10-minute delayed HPI provided satisfactory information for the detection and characterization of FHL. However, the lesion-to-liver contrast-to-noise ratio (CNR) or signal ratio obtained on 10-minute delayed hepatocyte phase images was significantly lower than that of the conventional imaging with 20-minute delay [13–16].

HPI is usually obtained with a T1-weighted (T1W) fat-suppressed (FS) three-dimensional (3D) gradient echo (GRE) sequence with a low flip angle (FA) ranging from 10° to 15°. Generally, a low FA has been used in nonspecific extracellular gadolinium chelate-enhanced dynamic T1W 3D GRE sequence, which increases liver-to-spleen CNR and allows shortening of TR and short acquisition time [17]. However, with the use of high FA (30–35°) in HPI after injection of gadoxetic acid has been shown to improve both lesion-to-liver CNR and FHL detection in many studies [18–21], since the high FA intensifies T1-weighting [19].

On a previous study [22], using 5 minute delayed HPI with 30° flip angle showed statistically increased CNR in hepatocellular carcinoma (HCC), but not in the metastatic lesions, compared to 20 minute delayed HPI with 10° flip angle. Therefore, 5 minute delayed imaging seemed not to be sufficient enough to replace 20 minute delayed imaging for detection of metastatic lesions of the liver.

Therefore, the purpose of this study was to compare the lesion-to-liver CNR and lesion detection sensitivity of a HPI protocol with a 10-minute delay and a 30° FA (10min-FA30) with those of a standard HPI protocol with a 20-minute delay and a 10° FA (20min-FA10) in patients with liver metastases. Our motivation for this comparison was to determine whether the 10min-FA30 protocol would be superior to the 20min-FA10 protocol with respect to lesion-to-liver CNR, detection sensitivity and reduce the delay time.

## Materials and Methods

The Gangnam Severance Hospital institutional review board (IRB) approved this retrospective study and written informed consent was waived. Patient records were anonymized and de-identified prior to analysis.

### Study Population and Standard reference

From March 2013 to July 2014, 93 consecutive patients with known or suspected liver metastasis from colorectal cancer underwent gadoxetic acid-enhanced MRI examination. Ten patients were excluded from our study because the final diagnosis was not available due to the lack of a reference standard. Of the remaining 83 patients, 78 had confirmed liver metastases and the remaining five had either benign lesions (n = 2) or no FHL (n = 3). To prevent the reviewing radiologists from assuming that all patients had liver metastasis, the five patients with benign or no FHL were also included. Therefore, a total of 83 patients (45 men, 38 women; mean age, 58 years; range, 29–81 years) with 248 liver metastases and 78 benign FHLs were analyzed. The patients had no underlying diffuse or cirrhotic liver disease. The primary malignancies of the patients presenting with liver metastases were colon cancer (n = 46) and rectal cancer (n = 37).

Among the 78 benign lesions (70 cysts, 7 hemangiomas, and 1 focal eosinophilic necrosis), one hemangioma was diagnosed pathologically by surgery. The other benign lesions were diagnosed based on laboratory findings, typical characteristic imaging criteria [18–23], and their non-progressive appearance in size on follow-up imaging studies and in examinations that took place before the study period.

Among the 248 metastatic tumors, 78 lesions were diagnosed pathologically (73 by resection and 5 by biopsy). The remaining 170 metastases in patients with the aforementioned primary malignancies were diagnosed based on the imaging findings that were similar to those of biopsy-proven metastatic tumors and their progressive appearance on follow-up imaging studies.

### Image Acquisition

All MRI examinations were performed with a 1.5 T scanner (Magnetom Avanto, a TIM system; Siemens Medical Solutions, Erlangen, Germany) using a 16-channel torso phased-array coil centered over the liver. The sequence protocol consisted of: breath-hold (BH) two-dimensional (2D) axial and coronal half-Fourier acquisition single-shot turbo spin-echo (HASTE); BH 2D axial GRE T1-weighted images with 2-point Dixon reconstructions; pre-contrast and dynamic 3D axial FS T1W volumetric interpolated breath-hold examination (VIBE) during the arterial, portal venous, 3- and 5-minute delayed dynamic phase; BH 2D axial T2W turbo spin-echo images, 10-minute delayed BH 3D axial FS T1W VIBE imaging; navigator-echo triggered FS 2D axial diffusion-weighted images using prospective acquisition correction; 20-minute delayed BH 3D axial FS T1W VIBE images.

Post-contrast MRI examination were performed with an intravenous infusion of 0.025 mmol/kg of gadoxetic acid (Primovist, Bayer Schering Pharma, Berlin, Germany) at a rate of 1 mL/s, followed by a 20 mL saline flush at the same injection rate. Infusions were administered with a mechanical power injector (MedRad Spectris Solaris EP, Medrad, Indianola, PA, USA) through a 20-gauge catheter inserted into an antecubital vein.

During 10-minute delayed phase imaging, 3D axial FS T1W VIBE images were acquired using a 30° FA. The other 3D axial FS T1W VIBE images (dynamic and 20-minute delayed imaging) were acquired using 10° FA. The 3D VIBE sequence parameters were as follows: TR 5.1 msec, TE 2.4 msec, receiver bandwidth 300 Hz/pixel, matrix 256 x 179, parallel acceleration factor 2 using the GRAPPA algorithm, slice thickness 2.8 mm, k-space trajectory rectangular (FA 10°) and central (FA 30°) ordering, and acquisition time of 14–15 seconds at both 10° FA and 30° FA imaging.

### Quantitative Image Analysis

Each metastatic hepatic tumor was classified as either large (short axis ≥10 mm) or small (short axis <10 mm). Tumor diameter was measured on the 20-minute delayed HPI. Quantitative analysis was performed by a coordinating radiologist who also attended in the confirmation of hepatic lesions. The signal intensities of each metastatic tumor and the surrounding normal liver parenchyma were measured on two imaging sets (10min-FA30 and 20min-FA10). Image noise was defined as the standard deviation of background signal intensity anterior to the liver and outside of the body. The measurements were performed three times, and the mean value was used for lesion-to-liver CNR calculation as follows: CNR = (SI_Liver_—SI_Tumor_)/SD_Noise_, where SI_Liver_ = mean signal intensity of the liver parenchyma, SI_Tumor_ = mean signal intensity of metastatic tumor, and SD_Noise_ = mean standard deviation of the background.

### Qualitative Image Analysis

Two radiologists who had 17 years and 8 years of experience in the MR imaging of the liver independently and randomly evaluated the two imaging sets (10min-FA30 and 20min-FA10). Images were analyzed 4–6 weeks apart to avoid recall bias. Moreover, individual sequences were randomly assigned to either the first or second reading. All images were assessed on a picture archive and communication systems workstation (Centricity RA1000, GE Healthcare, Milwaukee, USA). The images were adjusted to an optimal window setting for each case.

The radiologists evaluated the presence of hepatic lesions on each image based on the following four-point confidence scale: 1 = definitely absent (no identifiable lesion), 2 = probably absent (questionable), 3 = probably present, 4 = definitely present. All evaluated lesions were marked with arrows and numbers, and the resultant images were saved digitally on the workstation. A coordinating radiologist with 4 years of experience in liver MRI, who was not involved in the qualitative reading session, matched MRI findings to those of the reference standard.

### Statistical Analysis

All statistical analyses were performed using dedicated statistical software (SPSS 12.0; SPSS, Chicago, IL, USA). Sensitivities were calculated for the detection of all FHLs. Confidence scores of 1 and 2 were regarded as negative for the presence of a FHL, whereas confidence scores of 3 and 4 were considered as positive for the presence of a FHL. The sensitivities of the FHL detection on both 10min-FA30 and 20min-FA10 were compared using a Wilcoxon signed-rank test. Lesion-to-liver CNRs of large metastatic tumors (short axis ≥ 10 mm) were compared between groups by paired Student’s t-test. The kappa statistics was used to assess inter-reader agreement with respect to scoring and was interpreted according to the guidelines of Landis and Koch [23]. Significant differences were defined as those with p values less than 0.05.

## Results

**Fig 1:**
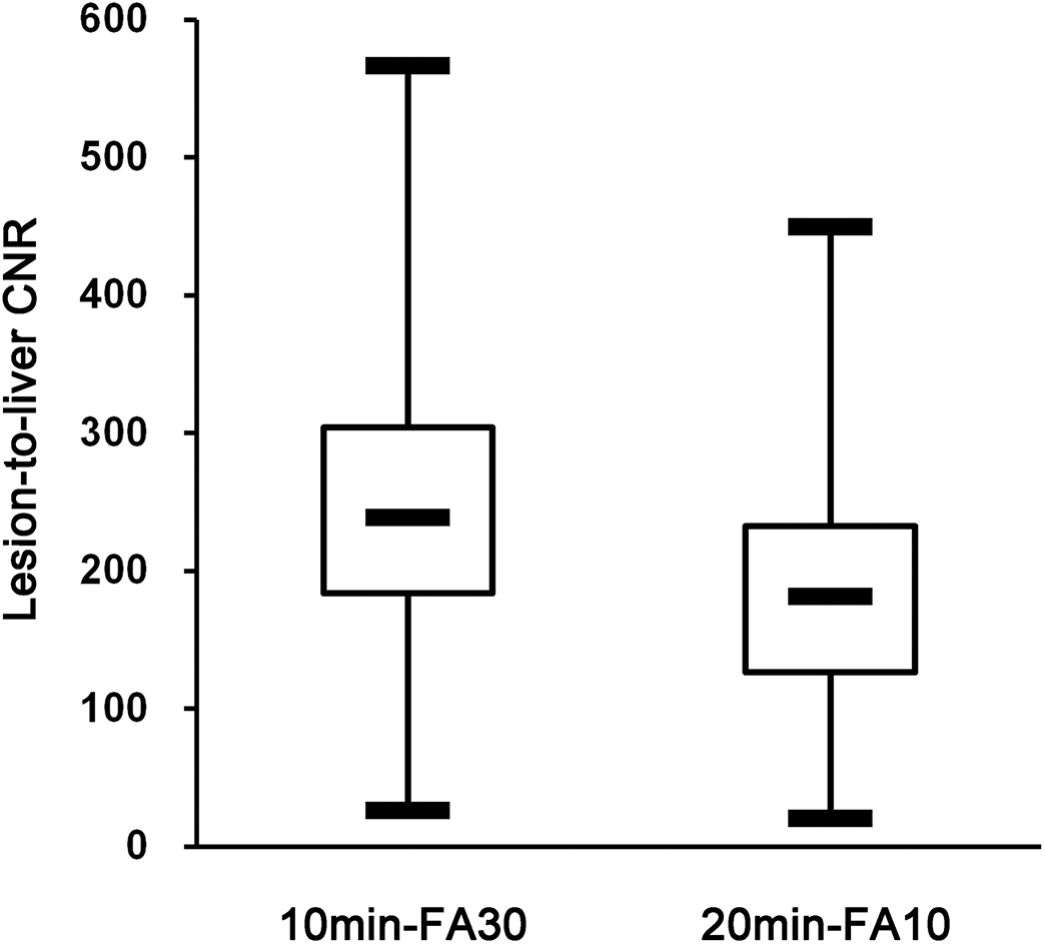
Lesion-to-liver contrast-to-noise ratio (CNR). Box-and-whisker plots showing the median (middle line of each box), quartiles (top and bottom lines of each box), and upper and lower adjacent (upper and lower whiskers for each box) values of the lesion-to-liver contrast-to-noise ratio (CNR). Liver metastases were imaged according to two different protocols: 10 minute delayed hepatocyte phase imaging with a 30° flip angle (10min-FA30) and 20 minute delayed hepatocyte phase imaging with a 10° flip angle (20min-FA10). The mean CNR for FHLs imaged with the 10min-FA30 protocol was significantly higher than that of FHLs imaged with the 20min-FA10 protocol.

**Fig 2:**
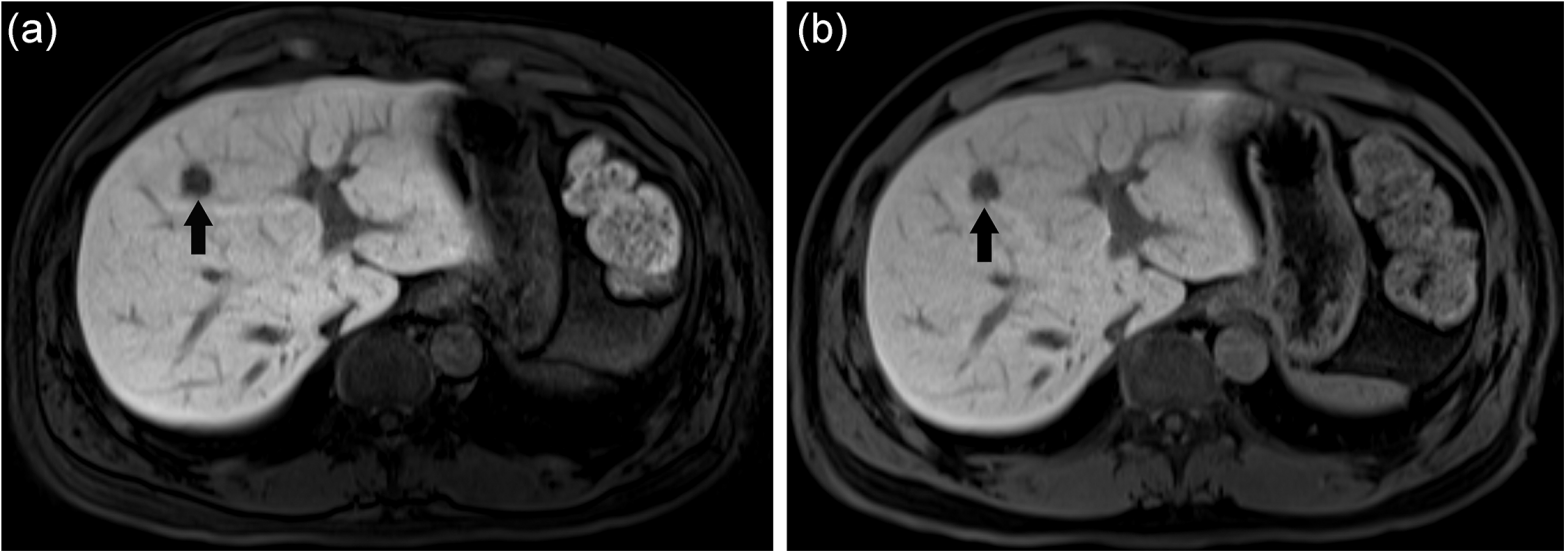
43-year-old male with pathologically proven liver metastasis from rectal cancer. Gadoxetic acid-enhanced T1-weighted MR hepatocyte phase images taken with a 10-minute delay and a 30° flip angle (10min-FA30) (a) and a 20-minute delay and a 10° flip angle (20min-FA10) (b). The liver metastasis in segment 4 showed low signal intensity related to liver parenchyma on both images (dark arrows). The lesion-to-liver CNR of the 10min-FA30 image (190.3) was superior to that of the 20min-FA10 image (137.3). Two different radiologists each gave the 10min-FA30 and 20min-FA10 images a mean subjective score for focal hepatic lesion presence of 4.0.

Of the 248 metastatic tumors, 202 lesions with a short axis longer than 10 mm were quantitatively measured. The other 46 lesions, smaller than 10 mm, were not quantitatively analyzed. The mean CNR for liver metastases imaged with the 10min-FA30 (248.5 ± 101.6) was significantly higher than that of the metastases imaged with the 20min-FA10 (187.4 ± 77.4) (p < 0.001). Additionally, the mean CNR difference between the two image groups was 61.2 ± 56.8 (Fig 1). Moreover, 186 tumors out of 202 liver metastases with short axis ≥ 10 mm had a higher CNR with the 10min-FA30 protocol (Fig 2), whereas the other 16 tumors had a higher CNR with the 20min-FA10 protocol.

The FHL detection sensitivities of the two imaging protocols are provided in Table 1. No significant differences were observed between two imaging protocols with respect to sensitivity for either reader, irrespective of lesion malignancy; benignity; or FHL size. Two small false positive lesions were detected by reader A and one by reader B; both depicted on 20min-FA10 protocol. The interobserver agreement between the two readers was substantial (0.725).

**Table 1:** Detection Sensitivity of Focal Hepatic Lesion for the Two Readers on 10-minute Delayed Hepatocyte Phase Imaging using a 30° Flip Angle (10min-FA30) and 20-minute Delayed Hepatocyte Phase Imaging using a 10° Flip Angle (20min-FA10).

Delayed time & FA	Reader A	Reader B
All lesions		
10min-FA30	98.5% (321/326; 0)	96.9% (316/326; 0)
20min-FA10	98.8% (322/326; 1)	96.9% (316/326: 2)
P value	P = 0.564	P > 0.999
Large lesions		
10min-FA30	100% (247/247; 0)	99.2% (245/247; 0)
20min-FA10	100% (247/247; 0)	99.2% (245/247; 0)
P value	P > 0.999	P > 0.999
Small lesions		
10min-FA30	93.7% (74/79; 0)	89.9% (71/79; 0)
20min-FA10	94.9% (75/79; 1)	89.9% (71/79; 2)
P value	P = 0.564	P > 0.999
Malignant lesions		
10min-FA30	99.2% (246/248)	97.6% (242/248)
20min-FA10	98.8% (245/248)	96.4% (239/248)
P value	P = 0.317	P = 0.180
Benign lesions		
10min-FA30	96.2% (75/78)	94.9% (74/78)
20min-FA10	98.7% (77/78)	98.7% (77/78)
P value	P = 0.157	P = 0.083

Data in parentheses are true-positive lesions/all focal hepatic lesions and number of false-positive lesions. Large lesions are focal hepatic lesions 10 mm or greater in short axis. Small lesions are focal hepatic lesions less than 10 mm in short axis.

## Discussion

To achieve the maximum hepatic parenchymal enhancement in gadoxetic acid enhanced MRI, it is important to wait for the functioning hepatocytes to take up a sufficient amount of gadoxetic acid [14, 24]. Based on preliminary evaluations of gadoxetic acid, a delay time of 20 minutes after contrast injection has been proposed and used for HPI [2–4]. Since the introduction of the 20-minute delay, most MRI studies using gadoxetic acid have used this protocol [5, 9–11, 25–30]. However, considering recent development in MR imaging and clinical application, a delay time of 20 minutes may no longer be necessary. Decreasing this delay time would potentially improve throughput and be more economical [16]. In addition, a decreased delay time would improve patient discomfort during the MRI procedure.

Many groups have investigated to reduce the delay time for the hepatocyte phase in gadoxetic acid-enhanced MRI [13–16, 31]. Although early reports found that a 20-minute delay was required to achieve adequate HPI [2, 4, 16], later studies found that the accuracy of FHL and liver metastasis detection was comparable between hepatocyte phase images obtained with either a 10-minute delay or a 20-minute delay [13–16]. However, the liver parenchymal enhancement or lesion-to-liver signal ratios were shown to be higher when a 20-minute delay was used compared with a 10-minute delay [2, 4, 13, 16]. In addition, one recent report showed that liver parenchymal enhancement, CNR and SNR of hepatocyte phase images acquired after a 10-minute delay were either greater than or similar to those acquired after a 20-minute delay in patients with normal liver function [15].

T1-weighted GRE sequence uses small flip angle and short repetition time value to reduce the total scan time. Due to the relatively short repetition time, longitudinal magnetization is incompletely recovered at the relatively short repetition time. This effect is more evident for FHLs without gadoxetic acid uptake, which has a longer T1 relaxation time, compared with enhanced liver parenchyma, which has a shorter T1 relaxation time. This difference in residual longitudinal magnetization is amplified by increasing FA. Thus, increasing the FA also improves T1-weighting and provides a greater contrast between FHLs without gadoxetic acid uptake and enhanced liver parenchyma. Therefore, increased FA has been proposed to yield a better lesion-to-liver CNR and to improve FHL detection [19, 20]. Based on this hypothesis we tried to use higher FA to reduce the delayed time for HPI.

From previous reports, the mean lesion-to-liver CNR or contrast ratio on hepatocyte phase images obtained with a high FA (30–35°) were significantly higher than those obtained with a low FA (10–12°), with delays of 5, 10, 15, and 20 minutes [18, 20]. The mean lesion-to-liver contrast ratio of images acquired with a 30° FA after a 10-minute delay (3.96 ± 1.66) was higher than that of images acquired with a 10° FA and a 20-minute delay (2.27 ± 0.62), although statistical analysis was not performed. This result is in agreement with our results, as we found that the mean CNR of metastatic tumors on 10min-FA30 images (248.5 ± 101.6) was higher than that of metastatic tumors on 20min-FA10 images (187.4 ± 77.4).

Previously, a study to shorten the delayed time from 20 minutes to 5 minutes was conducted with 30° flip angle in gadoxetic acid-enhanced MRI. On the study, 5 minute delayed HPI with 30° FA showed increased lesion-to-liver CNR for HCC, but did not show sufficient results for metastatic lesions compared to the conventional 20 minute delayed HPI with 10° FA. The different results between the HCC and metastatic lesions are not clearly understood. However, for the evaluation of the liver metastasis, at least 10 minute delay seems to be required.

Using 3.0T MR imaging, no significant difference in the sensitivity of liver metastasis detection was observed between procedures using a standard 10–13° FA and a delay of either 10 or 20 minutes [13, 14]. One previous study, which used a higher FA [20], found that the detection sensitivities for FHLs (including metastases) with a protocol using a 30° FA and a 10-minute delay (mean 96.5%) were higher than those obtained using a 10° FA and a 20-minute delay (mean 91.0%); however, no statistical analysis was performed. Our study also found no significant difference in the sensitivity of detection of FHLs between the 10min-FA30 and 20min-FA10 protocols, irrespective of lesion size or malignancy.

While using a higher FA to improve lesion-to-liver CNR, it also increases the energy deposition in patient’s tissue by radiofrequency field, which is expressed as the specific absorption rate (SAR). The SAR increases with field strength, radiofrequency power, duty cycle, transmitter-coil type, and body size. Moreover, the SAR is proportional to the square of the FA. Therefore, increasing the FA from 10° to 30° results in a nine-fold increase in the SAR. Although this increase is not usually problematic at 1.5T, it can be challenging for 3.0T MR imaging, where the baseline SAR is already quadrupled compared with that of 1.5 T imaging [19, 20].

There were some limitations in this study. Firstly, our study was retrospective and all of the FHLs could not all be diagnosed pathologically. Nevertheless, an experienced abdominal radiologist reviewed all clinical and follow-up information for the final diagnosis in each case that lacked pathologic correlation. Secondly, the CNR of small FHLs with short axis <10 mm was not analyzed. To minimize the effect of partial volume artifacts, signal intensity was only measured for large lesions with a short axis ≥ 10 mm. Third, in this a HPI protocol with a 30° FA and a 20-minute delay was not analyzed because previous reports did not find any significant difference in FHL detection sensitivities for high or low FA imaging protocols with either a 15- or 20-minute delay [20]. Finally, none of the patients enrolled in this study had chronic hepatitis or cirrhosis. Varying degrees of chronic liver disease might influence the detection sensitivity of FHLs or lesion-to-liver CNR on HPI. Thus, further studies are needed for this matter.

In conclusion, the gadoxetic acid-enhanced MRI protocol for HPI with a high FA (30°) and a 10-minute delay yielded a higher lesion-to-liver CNR compared with a standard protocol with a low FA (10°) and a 20-minute delay. In addition, no significant difference was observed between the two protocols regarding lesion detection sensitivity. These results may suggest that the 10min-FA30 protocol can replace the 20min-FA10 protocol with better diagnostic performance in the detection of liver metastases and also saves 10 minutes in selected patients.

## Supporting Information

S1 File Attached files are data of the sensivity and lesion-to-liver CNR. (XLSX)
